# What Proportion of New Tuberculosis Patients Has a History of Household Tuberculosis Exposure? A Cross-Sectional Study from Udupi District, South India

**DOI:** 10.3390/tropicalmed4040133

**Published:** 2019-11-01

**Authors:** Chidananda Sanju SV, Nikhil Srinivasapura Venkateshmurthy, Divya Nair, Vrinda Hari Ankolekar, Ajay MV Kumar

**Affiliations:** 1 District Tuberculosis Control Office, Behind District Health Office, Ajjarakadu, Udupi, Karnataka 576104, India; 2 Public Health Foundation of India, Gurgaon 122002, India; nikhil.sv@phfi.org; 3 Harvard TH Chan School of Public Health, Boston, MA 02115, USA; 4 The INCLEN Trust International, New Delhi 110020, India; divya.nair@inclentrust.org; 5 Manipal Academy of Higher Education, Kasturba Medical college, Manipal 576104, India; vrindahari@rediffmail.com; 6 International Union against Tuberculosis and Lung Disease (The Union), 75020 Paris, France; AKumar@theunion.org; 7 The Union South-East Asia Office, New Delhi 10001, India; 8 Yenepoya Medical College, Yenepoya (Deemed to be University), Mangaluru 575018, India

**Keywords:** tuberculosis preventive therapy, isoniazid preventive therapy, household contacts, operational research, SORT IT

## Abstract

While tuberculosis (TB) preventive therapy among household contacts is effective at an individual level, its population-level impact on reducing TB incidence has been unclear. In this study, we aimed to assess, among the new tuberculosis patients started on treatment between 1 October, 2018 and 30 June, 2019 in the public health facilities of Udupi district (South India): i) the proportion with a ‘history of household TB exposure’ and ii) sociodemographic and clinical factors associated with it. We conducted a cross-sectional study involving record review and patient interviews. Of 565 TB patients, 273(48%) were interviewed. Of them, 71(26%, 95% CI: 21%–32%) patients had a ‘history of household TB exposure (ever)’ with about half exposed in the past five years of diagnosis. Considering a new TB case as a proxy for incident TB, and ‘history of household TB exposure’ a proxy for household transmission, and assuming 100% effectiveness of preventive therapy, we may infer that a maximum of 26% of the incident cases can be prevented by giving preventive therapy to all household contacts of TB patients. In multivariable analysis, females and tobacco users had a significantly higher prevalence of household TB exposure. If there are resource constraints, these subgroups may be prioritized.

## 1. Introduction

Tuberculosis (TB) is the leading infectious cause of death worldwide ranking above human immunodeficiency virus (HIV). In 2017, 10 million people fell ill with TB and 1.6 million people died due to it. While TB incidence has been decreasing over the years, the rate of decrease has been modest. At the current rate of decline, we will not be able to end TB as envisaged by the World Health Organization (WHO) [1] and the United Nations Sustainable Development Goals [2].

Ending TB requires focus to prevent TB. Since there is no effective vaccine to prevent TB, identification, and treatment of latent TB infection and halting the progression to active TB disease is the cornerstone of prevention. The WHO targets the provision of 30 million preventive treatments between 2018 and 2022. Traditionally, preventive therapy was recommended only among household contacts (aged < 5 years old) and people living with HIV (PLHIV). In 2018, the WHO in its consolidated guidance on programmatic management of LTBI recommended extending preventive therapy to all household contacts (without TB) including children ≥ 5 years old, adolescents, and adults [3].

While there is strong evidence that preventive therapy among household contacts is effective in preventing TB disease at an individual level, its effect on reducing TB incidence in the population has been unclear. A systematic review of published studies between 1929 and 2015 showed that only a small proportion (11%–14%) of all infections was attributable to household exposure, indicating that much of the transmission occurs outside of the household [4]. 

We hypothesize that the proportion of TB disease attributable to household transmission varies based on the burden of TB in the population. In high incidence settings, the chance of having contact with TB outside the household is high and hence the contribution of community transmission is high. In low incidence settings, the chance of community transmission is relatively lower and hence, the proportion of TB disease attributable to household transmission is likely to be high. This also means that implementing a strategy of preventive therapy among all household contacts (including adults) will have a greater impact on TB incidence in such settings. 

There is limited evidence on this issue from India. A 1968 cohort, in a high TB burden setting in India, reported that only 8.5% of all incident TB patients had a household member with TB, a proxy for the ‘proportion of TB attributable to household transmission’ [5]. 

The TB epidemic has evolved over the past five decades in India. The burden of TB is declining, especially in South India. In this context, the community-level transmission might have declined and the proportion of incident TB attributable to household TB exposure might have increased. This is in line with results of a more recent study (2016–2017) conducted in India, which shows that 25% of new sputum-positive TB patients had a history of household contact with tuberculosis [6]. 

In this study, we aimed to assess among the new TB patients started on treatment in a district in South India, (i) the proportion with a ‘history of household TB exposure’ along with the timing of exposure and (ii) sociodemographic and clinical factors associated with it.

## 2. Materials and Methods

### 2.1. Study Design

This was a cross-sectional study involving record review and patient interviews.

### 2.2. Setting

**Figure 1:**
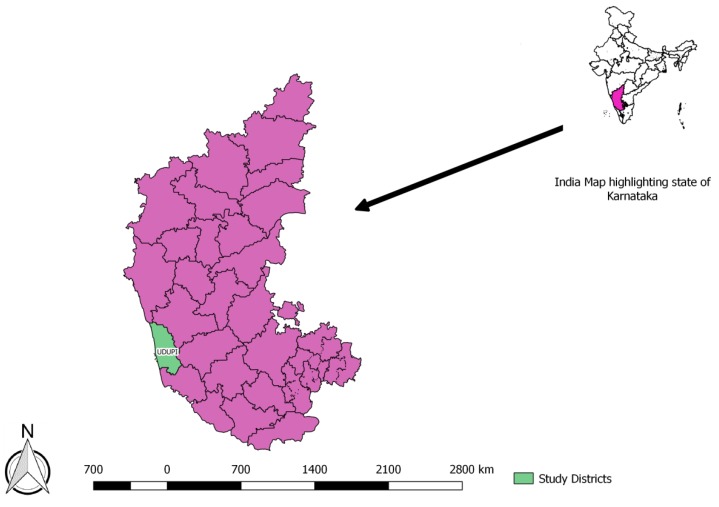
Depicting the Udupi district, Karnataka, India.

Karnataka is a state in the southern part of India with a total of 30 districts. The study was conducted in Udupi district (Figure 1) which is located in the coastal region of Karnataka and has a population of 1.2 million. Karnataka state has a TB prevalence of 180/100,000 population whereas that in Udupi district is 66/100,000 [7]. There are 61 primary health centres, 6 community health centres, 2 block-level hospitals, and a district hospital. There are 54 sputum microscopy centres wherein diagnostic testing for tuberculosis is available. Currently, preventive therapy is offered to only child contacts (<6 years) of smear-positive tuberculosis patients and PLHIV. Isoniazid at a dose of 10 mg/kg/day is recommended for duration of six months among children, while this is 300 mg per day among adults. The drugs are collected by the patients once a month and self-administered.

### 2.3. Study Population:

We included all new tuberculosis patients started on treatment between 1 October, 2018 and 30 June, 2019 in the public health facilities of Udupi district. Patients who were available during home visits by the field staff and consented to participate in the study were interviewed. Due to constraints in time and funding, we could not make additional visits to reach the patients who were unavailable during the first visit.

### 2.4. Sample Size

Assuming the prevalence of household tuberculosis exposure of 20%, and absolute precision of 5%, the sample size required was calculated to be 246. We expected to achieve the sample size during the study period.

### 2.5. Data Variables, Sources of Data, and Data Collection

Data was collected from two sources—TB treatment cards and interviewer-administered questionnaire. Socio-demographic and clinical variables were extracted from the TB treatment card. For the same patients, data about the history of household TB exposure, the timing of exposure and relationship with the source case were collected in the interviews. Patients unable to recollect the information were urged to enquire their family members and report back about the household TB exposure. Data on ownership of assets and key housing characteristics (water supply, type of toilet and whether it is shared and cooking fuel) was also collected to calculate wealth index. 

Interviews were conducted by trained staff (Senior Treatment Supervisors and TB Health visitors) using a pre-tested, structured questionnaire. We used a mobile-phone-based, quality-assured, electronic tool (EpiCollect5, Imperial college of London, UK) to capture the data.

For patients who could not be interviewed, we extracted data on baseline demographic and clinical characteristics from NIKSHAY, a case-based, web-based TB notification portal.

### 2.6. Analysis and Statistics

The analysis was conducted using EpiData version 2.2.2.186 (EpiData Association, Odense, Denmark) and Stata (version 15, Stata Corp LLC). Continuous variables were described using mean and standard deviation. Categorical variables were summarized as the proportion with 95% confidence interval. Principal Components Analysis was carried out to calculate the household wealth index. The proportion (with 95% CI) of new TB patients with a history of household TB exposure was calculated. Association between the history of household TB exposure and various socio-demographic factors was measured using prevalence ratio (with 95% CI). We performed Poisson regression to calculate adjusted prevalence ratio and identify factors independently associated with the outcome. This was done primarily to identify the possible subgroups with a higher prevalence of household TB exposure.

### 2.7. Ethics Approval

Ethics approval was obtained from the Ethics Advisory Group of the International Union Against Tuberculosis and Lung Disease, Paris, France (Number 129/18). Informed written consent was obtained from all the participants. Formal approval to access programme data was obtained from the State TB officer.

## 3. Results

Of the 565 TB patients started on treatment during the study period, 273 (48%) were available during home visits by the field staff and consented to be interviewed. Since the response rate was lower than expected, we compared the interviewed and non-interviewed patients to examine if there were any differences in baseline characteristics (Table 1). The groups were similar except for HIV status and site of TB.

Of the 273 interviewed, 66% were males and the mean (SD) age was 43 (16) years. A total of 236 (86%) patients resided in rural areas and 155 (57%) patients were undernourished (BMI < 18.5 kg/m^2^). About one in five patients reported alcohol use (*n* = 57, 20%) and tobacco use (*n* = 52, 19%).

There were a total of 1079 contacts living in the households of patients interviewed—130 below the age of six years (mean of 0.5) and 949 aged six years and above (mean of 3.5).

Of the 273 interviewed, 71 (26%, 95% CI: 21%–32%) patients had a ‘history of household TB exposure (ever)’. Microbiologically confirmed TB patients, pulmonary TB patients, and people with a history of current tobacco and alcohol use had a higher prevalence of household TB exposure in unadjusted analysis **(Table 2)**. In multivariable analysis, we found that female sex and tobacco use were significantly associated with household TB exposure.

Of the 71 TB patients with a history of household TB exposure, information on the time of exposure was not available for one-third of respondents. Among the rest, 45% reported a history of exposure within the past five years **(Table 3**).

Parents (41%) were reported as the most frequent source of exposure followed by a spouse (16%), grandparents (14%), siblings, and others (Table 4).

## 4. Discussion

This is one of the few studies from India which estimated the prevalence of ‘household TB exposure’ among new TB patients, thus adding to the national evidence on this issue. We found that one in four patients had household TB exposure. This is thrice higher than that reported from a 1968 cohort (8.5%) and similar to the 2016-17 cohort (25%) reported from India [5,6]. Nearly half of the TB patients reported exposure within the past five years with parents being the most common source of exposure. 

Considering a new TB case as a proxy for an incident case and ‘history of household TB exposure’ as a proxy for household transmission, we may infer that a maximum of 26% of the incident cases can be prevented by giving preventive therapy to all household contacts of TB patients. Please note that this assumption may not be entirely correct because we have not done a transmission study using molecular epidemiology techniques. Also, preventive therapy may not be 100% effective as assumed here and may depend on various factors such as efficacy of the treatment regimen used, coverage, and completion rates. However, this might be the maximum impact that can be expected out of a strategy of providing preventing therapy to all household contacts. It is also possible that some of the study participants might not be aware of a case of TB in their household, especially if the source of exposure had occurred more than ten years ago. If that is the case, we might be underestimating the impact here.

We found that the prevalence of ‘household TB exposure’ was higher among certain patient sub-groups—like females and those with a history of tobacco use. This information is useful for prioritizing programme resources. If there are resource constraints and the national TB programme is planning for phased implementation, then targeting these subgroups among household contacts is likely to be more effective. This decision requires careful consideration of other aspects such as individual risk of developing TB in such sub-groups and cost-effectiveness data.

We found that on an average, there were four contacts in the household per TB patient—of whom nearly 90% are contacts aged six years and above. Currently, only children less than six years are targeted for preventive therapy in India. So, if we were to roll out preventive therapy to all contacts, it will add a substantial workload to the current efforts. This information is useful for the national TB programme in calculating the drug requirements and planning the drug procurement and supply chain management, one of the key implementation challenges noted globally.

The challenges of implementing preventive therapy are well documented. First, the uptake of preventive therapy has been poor globally and in India. In 2017, globally only 36% of PLHIV newly enrolled in HIV care and 23% of under-five child contacts received preventive therapy. This is even lower at 10% in India among both PLHIV and under-five children. Second, the completion rates of preventive therapy are low. Reasons for this include the long duration of treatment for 6–9 months and adverse drug effects. The WHO now recommends many shorter regimens including 12 weekly doses of isoniazid and rifapentine (3HPregimen) [3]. Several studies indicate that 3HP regimen has higher rates of completion, is more effective and safer compared to 6 months isoniazidmonotherapy [8,9,10,11]. 

The study has several strengths. The data on household TB exposure is not systematically collected in the programme and hence, we interviewed the patients. The data capture was done electronically, using the freely available data collection platform (EpiCollect5) to reduce errors and improve the accuracy of collected data. We used existing Revised National TB Control Programme staff to carryout data collection without entailing any additional costs. Apart from the history of exposure, we have also collected information on the timing of the exposure and the relationship between the TB case and the source of exposure.

We had some limitations too. We had a less-than-expected response rate to the survey because of resource constraints. Thus, the data collectors could only visit the eligible participants once and could not make any further visits. Had they been able to visit once or twice more, the response rate could have been higher. We tried to mitigate this by comparing the characteristics of participants who were interviewed with those who were not. However, we were limited by the number of variables we could use to compare as the Nikshay portal contained only a select few characteristics. The patients not interviewed were more likely to be HIV-positive as compared to those interviewed. This may affect our overall estimate and it may not be possible to generalize the findings to HIV-positive TB patients. However, that may not have much policy relevance, because PLHIV are already eligible to receive preventive therapy whether they are a household contact or not. Non-responders were less likely to have pulmonary TB and had we interviewed everyone, the history of household TB exposure would have been marginally lesser than 26%. The other limitation was related to the fact that neither the study participants nor the source cases had a culture-based diagnosis and there was no molecular fingerprint to indicate that they belong to the same cluster. Hence, we cannot be certain that those with a history of household TB exposure indeed represent household transmission. We also could not validate, through patient records, if the source cases indeed had tuberculosis. Although, given that TB is a major life-event, it is unlikely that recall limitation might have played a role here. Finally, since the study was conducted just in one district of the state of Karnataka, this may have limited generalizability beyond the study area. We need more studies to inform policy.

In conclusion, our study sheds light on the crucial aspect of household TB exposure and its implications for preventive therapy. We found that 26% of new TB patients had a history of ‘household TB exposure’. As a corollary, this means that we may expect to achieve a maximum of 26% reduction in TB incidence if we roll out the strategy of preventive therapy to all household contacts. This information will be helpful for policymakers to make an informed decision.

## Figures and Tables

**Table 1:** Comparison of clinical and demographic characteristics of new tuberculosis (TB) patients started on treatment at Udupi district, Karnataka, India, from October 2018 to June 2019 who were interviewed and not interviewed.

Characteristic	Interviewed		Not Interviewed		*p*-Value
	**N**	**(%)#**	**N**	**(%)#**	
**Total**	**273**	**(100)**	**292**	**(100)**	
**Gender**					0.63
Male	180	(66)	198	(68)	
Female	93	(34)	94	(32)	
**Age (years)**					0.09
<15	2	(1)	5	(1)	
15–29	63	(23)	46	(16)	
30–44	81	(30)	78	(27)	
45–59	81	(30)	99	(34)	
≥60	46	(16)	64	(22)	
**HIV status**					<0.01
Reactive	6	(2)	48	(16)	
Non-reactive	263	(96)	229	(79)	
Not recorded	4	(2)	15	(5)	
**Diabetes status**					0.11
Yes	41	(15)	34	(12)	
No	195	(71)	200	(68)	
Unknown	37	(14)	58	(20)	
**Basis of TB diagnosis**					0.09
Microbiologically confirmed	212	(78)	209	(72)	
Clinically diagnosed	61	(22)	83	(28)	
**Site of the disease**					0.04
Extra pulmonary	60	(22)	86	(30)	
Pulmonary	213	(78)	206	(70)	
**Current Tobacco use**					0.13
Yes	52	(20)	62	(21)	
No	217	(80)	230	(79)	

# The percentages may not add up to 100% as we have excluded patients with missing information in some variables.

**Table 2:** Clinical and socio-demographic factors associated with the history of household TB exposure among new TB patients started on treatment in Udupi district, Karnataka, India from October 2018 to June 2019.

Characteristics	Total	Household TB Exposure		PR	95% CI	aPR	95% CI
		N	(%)				
Total	273	71	(26)				
**Age in years**							
<30	66	22	(31)	Ref		Ref	
30–59	163	43	(26)	0.79	(0.52–1.21)	0.90	(0.50–1.64)
≥60	44	6	(14)	0.41	(0.18–0.93)	0.56	(0.21–1.44)
**Gender**							
Male	180	41	(23)	Ref		Ref	
Female	93	30	(32)	1.42	(0.95–2.11)	**2.51**	**(1.37–4.61)**
**Area of residence**							
Rural	236	58	(25)	Ref		Ref	
Urban	37	13	(35)	1.43	(0.87–2.34)	1.26	(0.67–2.35)
**Household wealth quintile**							
1 (lowest)	55	21	(38)	1.83	(0.98–3.43)	1.42	(0.64–3.19)
2	56	18	(32)	1.54	(0.80–2.96)	1.40	(0.62–3.19)
3	57	14	(25)	1.18	(0.59–2.37)	1.00	(0.42–2.33)
4	52	7	(14)	0.65	(0.27–1.54)	0.54	(0.19–1.49)
5 (highest)	53	11	(21)	Ref		Ref	
**Type of TB**							
Microbiologically confirmed	202	61	(30)	**2.35**	**(1.23–4.47)**	2.05	(0.96–4.38)
Clinically diagnosed	70	9	(13)	Ref		Ref	
**Site of disease**							
Pulmonary	209	61	(29)	**1.87**	**(1.02–3.43)**	NA	
Extra pulmonary	64	10	(16)	Ref			
**HIV status**							
Reactive	6	1	(17)	0.63	(0.10–3.83)	0.70	(0.09–5.23)
Non-reactive	266	70	(26)	Ref		Ref	
**Diabetes status**							
Yes	39	7	(18)	0.66	(0.33–1.33)	0.90	(0.39–2.09)
No	234	64	(27)	Ref		Ref	
**Current tobacco use**							
Yes	52	25	(48)	**2.32**	**(1.58–3.40)**	**1.99**	**(1.06–3.78)**
No	217	45	(21)	Ref		Ref	
**Current alcohol use**							
Yes	57	22	(39)	**1.78**	**(1.17–2.69)**	1.61	(0.83–3.17)
No	212	46	(22)	Ref		Ref	
**Body mass index (kg/m^2^)**							
Undernourished (<18.5)	155	43	(28)	1.12	(0.73–1.70)	1.12	(0.73–1.70)
Normal (18.6–24.9)	105	26	(25)	Ref		Ref	
Overweight/Obese (≥25.0)	12	2	(17)	0.67	(0.18–2.49)	0.67	(0.18–2.49)

PR—crude prevalence ratio; aPR—adjusted prevalence ratio; CI—Confidence Intervals; NA—Not applicable as these variables were not included in the multivariable model due to collinearity; Factors with *p-*value < 0.05 have their PR and 95% CI in bold font.

**Table 3:** Timing of exposure to the source case among the TB patients who had a history of contact, in Udupi district, Karnataka, India during October 2018–June 2019 (*n*= 71).

Time Since Exposure	Number	(%)
Within1 year	10	(14)
1–5 years	12	(17)
6–10 years	8	(11)
10 years or more	18	(25)
Unknown time of exposure	23	(33)

**Table 4:** The relationship with the source case among the TB patients who had a history of contact, in Udupi district, Karnataka, India during October 2018–June 2019 (*n* = 71).

Relationship with the Source Case	Number	(%)
Parent	29	(41)
Spouse	11	(16)
Grandparent	10	(14)
Offspring	6	(8)
Sibling	3	(4)
Others*	7	(10)
Relationship unknown	5	(7)

* Others include uncle, aunt, and brother-in-law.
